# Characterization of Contrast-Enhancing and Non-contrast-enhancing Multiple Sclerosis Lesions Using Susceptibility-Weighted Imaging

**DOI:** 10.3389/fneur.2019.01082

**Published:** 2019-10-18

**Authors:** Philipp Eisele, Katja Fischer, Kristina Szabo, Michael Platten, Achim Gass

**Affiliations:** ^1^Department of Neurology, Universitätsmedizin Mannheim, University of Heidelberg, Mannheim, Germany; ^2^Department of Neurology, Klinikum der Stadt Ludwigshafen, Ludwigshafen, Germany

**Keywords:** multiple sclerosis, lesions, MRI, susceptibility-weighted imaging, central vein

## Abstract

Susceptibility-weighted magnetic resonance imaging (MRI) (SWI) offers additional information on conventional MRI contrasts. Central veins can be identified within lesions, and recently, it has been suggested that multiple sclerosis (MS) lesions with slowly expanding demyelination, so-called smoldering lesions, can be identified by a phase rim surrounding the lesion. We analyzed post-contrast SWI in regard to intrinsic lesion characteristics in a cohort of MS patients. A total of 294 MS patients were evaluated using a 3-T MRI. A comprehensive MRI protocol was used including post-contrast SWI. Lesions of at least 5 mm in size were analyzed on conventional MRI and SWI with a structured reporting scheme with a focus on SWI lesion characteristics. A total of 1,323 lesions were analyzed: 1,246/1,323 (94%) were non-enhancing and 77/1,323 (6%) were contrast-enhancing (CE) lesions. In CE lesions, the following patterns were seen: contrast enhancement was nodular in 34/77, ring-shaped enhancement was present in 33/77, and areas of peripheral enhancement were present in 10/77. In CE lesions, an association with central veins was found in 38/77 (50%). In 75/1,246 (6%) non-enhancing lesions, a central dark dot in keeping with a central vein was seen, whereas 162/1,246 (13%) showed peripheral hypointense dots/rims, 199/1,246 (16%) showed scattered hypointense dots mainly within the lesion area, and in 374/1,246 (30%), no SWI hypointensity was detected. Furthermore, 436/1,246 (35%) lesions showed isointensity to the surrounding tissue and were not visible on SWI. SWI is able to offer additional aspects of MS pathology also when used after the application of a contrast agent. Veins connected to lesions, a potentially useful marker in the differential diagnosis of MS, were seen in about 50% of enhancing lesions. Susceptibility artifacts, suggested to mark the presence of myelin-laden macrophages and smoldering inflammation, were visible in 28% of lesions as hypointense dots in and in the periphery of the lesion. Given those results, SWI may provide practical useful additional information in the evaluation of the lesion status in MS patients.

## Introduction

Conventional magnetic resonance imaging (MRI) techniques play an important role in the diagnosis and monitoring of disease evolution in multiple sclerosis (MS). Review of MS lesion characteristics can provide insight into the acute and chronic pathology. Hyperintense lesions on T2-weighted (T2-w) images are thought to represent a heterogeneous pathology such as edema, gliosis, axonal loss, and demyelination (1). New T2 lesions on follow-up MRI are commonly employed in the search for a recent inflammatory disease activity. In addition, the presence of contrast enhancement on post-contrast T1-weighted (T1w) images and occasionally hyperintensity on diffusion weighted MRI can also indicate acute inflammatory changes (2–5). Recently, susceptibility-weighted imaging (SWI), a velocity-compensated three-dimensional gradient echo sequence (6), has been shown to be sensitive to iron on the form of ferritin, hemosiderin, and deoxyhemoglobin (7). It is sensitive to iron accumulation in the normal-appearing brain tissue, in lesions, and in the vessel walls of veins in MS (8). Iron is supposed to cause damage to oligodendroctyes and myelin by oxidative stress in the form of free radicals (9). Furthermore, areas of active myelin breakdown also show susceptibility effects in the form of dark dots or rims (10–12). The susceptibility effects in those lesions are due to the presence of iron-laden activated microglia/macrophages and reactive astrocytes at the lesion edge (6, 8–11, 13). These lesions have been suggested to represent areas of so-called smoldering inflammation (10, 11, 13). Furthermore, it has been demonstrated that these lesions may increase in size during longer observation periods. Such gradual slowly evolving increases in lesion size can also be identified when analyzing serial T1w MRI as it has recently been demonstrated (14). This type of lesion has also generated high clinical interest, as it is conceivable that it contributes to gradual clinical worsening in MS.

Commonly, SWI is acquired before contrast agent injection, and only a few studies evaluated contrast-enhancing (CE) lesions on SWI after contrast application (15, 16). We investigated characteristics of active CE and non-enhancing lesions detected on contrast-enhanced SWI.

## Materials and Methods

### Subjects

We performed a retrospective, cohort analysis of MRI in MS patients in our MS database over a 3-year interval. Inclusion criteria were diagnosis of MS according to the McDonald diagnostic criteria (17) and being at least 18 years of age. Exclusion criteria were presence of neurological conditions other than MS, cardiovascular or respiratory disease, contraindication to MRI, and pregnancy or breastfeeding.

### MRI Studies

All MRI studies were performed on a 3.0-T MR system [MAGNETOM Skyra, Siemens, Erlangen, Germany, 20-channel head coil, 50-cm field of view [FOV]]. A standardized protocol was used in all patients: (1) axial, coronal, and sagittal localizing sequences followed by axial oblique contiguous 5-mm slices aligned with the inferior borders of the corpus callosum; (2) T2-w images (TR 4,000 ms/TE 78 ms, FOV 220 mm, slice thickness 3 mm, voxel size 0.4 × 0.4 × 3.0 mm); (3) fluid-attenuated inversion recovery (FLAIR) images (TI 2,500 ms/TR 8,500 ms/TE 136 ms, FOV 220 mm, slice thickness 3 mm, voxel size 0.4 × 0.4 × 3.0 mm); (4) T1w images (TR 225 ms/TE 2.5 ms, FOV 220 mm, slice thickness 3 mm, voxel size 0.7 × 0.7 × 3.0 mm); (5) T2^*^-w images (TR 594 ms/TE 19.9 ms, FOV 220 mm, slice thickness 3 mm, voxel size 0.4 × 0.4 × 3.0 mm); and (6) identical to (4) T1w images 10 min after manual injection of single-dose contrast agent of 0.1 mmol/kg body weight (Dotarem, Guerbet) followed by (7) susceptibility-weighted imaging (TR 27 ms, TE 20 ms, FOV 220 mm, slice thickness 1.50 mm, voxel-size 0.9 × 0.9 × 1.5 mm). SWI and mini-IP images were generated automatically by the scanner software provided by the manufacturer.

To exclude a possible influence of the contrast agent on diagnostic image quality, vascular enhancement, and parenchymal signal alterations in 10 patients were additionally examined with SWI before and after contrast agent injection.

### Data Processing and Analysis

Image interpretation was performed on a standard picture archiving and communication system workstation. Two readers experienced in the diagnostic and clinical reading of MS studies examined MR images jointly. Readers were unaware of clinical data and patient identification information. A structured reporting scheme was used. For lesion identification, FLAIR images were used as the “gold standard.” Only brain lesions with a diameter >5 mm on axial slices entered further analysis.

With the use of post-contrast T1w images, lesions were classified as CE lesions (group 1) or non-enhancing lesions (group 2), according to previously published criteria (18). All lesions were further classified on T1w images as T1 hypointense (19) or T1 isointense. In a second step after lesion identification on conventional MR images, SW images were analyzed for the corresponding lesion characteristics. As CE is seen also on SW images, CE lesions in group 1 were categorized as follows: (1) ring-shaped CE lesions, (2) lesions with partial peripheral contrast enhancement, or (3) nodular homogenous contrast enhancement. All lesions of group 1 (CE lesions) and group 2 (non-enhancing lesions) were categorized in regard to hypointense features potentially visible on SW images: (i) lesions with central veins, (ii) lesions with peripheral hypointense dots/rims, (iii) lesions with scattered hypointense dots within the lesion, and (iv) isointense lesions not seen on SWI. These categories were employed similar to previously suggested lesion characteristics (10, 11, 13).

### Standard Protocol Approval

This study was approved by the local institutional review board and performed in accordance with the ethical standards laid down in the 1964 Declaration of Helsinki and its later amendments. Patient consent was waived owing to the retrospective nature of the study and the lack of patient interaction.

## Results

A total of 294 patients [226 women and 68 men; mean age 36 years (range 18–69 years)] were included in the study. Two hundred seventy-nine patients had relapsing–remitting MS, and 15 patients had secondary progressive MS. The median Expanded Disability Status Scale (EDSS) was 2.0 (range 0–7). Two hundred twenty-four patients were on best individually selected treatment with interferon-beta, glatiramer acetate, dimethyl fumarate, fingolimod, or natalizumab. Seventy-six patients had an acute relapse at the time of MRI. Forty-four patients showed CE lesions on post-contrast T1w images.

**Figure 1:**
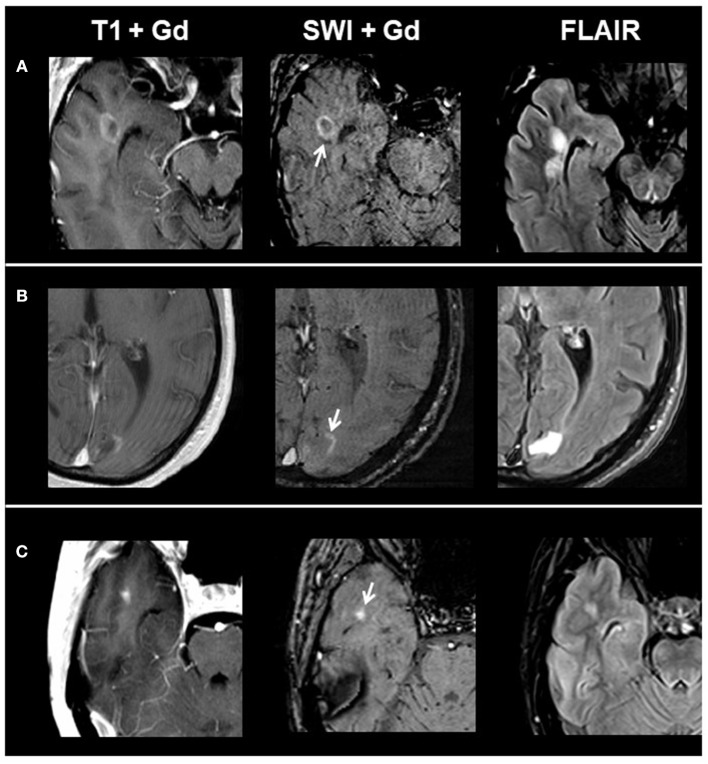
From left to right: representative post-contrast T1, post-contrast SWI, and FLAIR images of acute contrast-enhancing lesion subtypes. **(A)** Ring-shaped contrast enhancement, **(B)** peripheral contrast enhancement, and **(C)** homogenous contrast enhancement. SWI, susceptibility-weighted magnetic resonance imaging; FLAIR, fluid-attenuated inversion recovery.

Overall, we identified 1,323 lesions on conventional MRI: 77 CE lesions (group 1) and 1,246 non-enhancing lesions (group 2) (915 T1 hypointense lesions and 331 T1 isointense lesions). On T1 and SWI, 33/77 CE lesions presented with ring-shaped contrast enhancement, 10/77 with peripheral contrast enhancement, and 34/77 with homogenous contrast enhancement. When comparing post-contrast T1w and susceptibility-weighted images in respect to contrast enhancement, we found matching enhancement characteristics in all lesions. Figure 1 shows exemplary images of acute lesion subtypes identified on SWI, and Table 1 provides an overview on signal characteristics of CE lesions.

**Table 1:** MRI characteristics of contrast-enhancing lesions.

	**SWI lesions**	**Lesions associated with veins on SWI**	**T1 hypointense lesions**
Ring-shaped enhancing lesions on SWI	33	20 (60%)	33 (100%)
Peripheral enhancing lesions on SWI	10	4 (40%)	9 (90%)
Homogenous enhancing lesions on SWI	34	14 (40%)	34 (100%)
Total	77	38 (50%)	76 (98%)

*SWI, susceptibility-weighted magnetic resonance imaging*.

**Figure 2:**
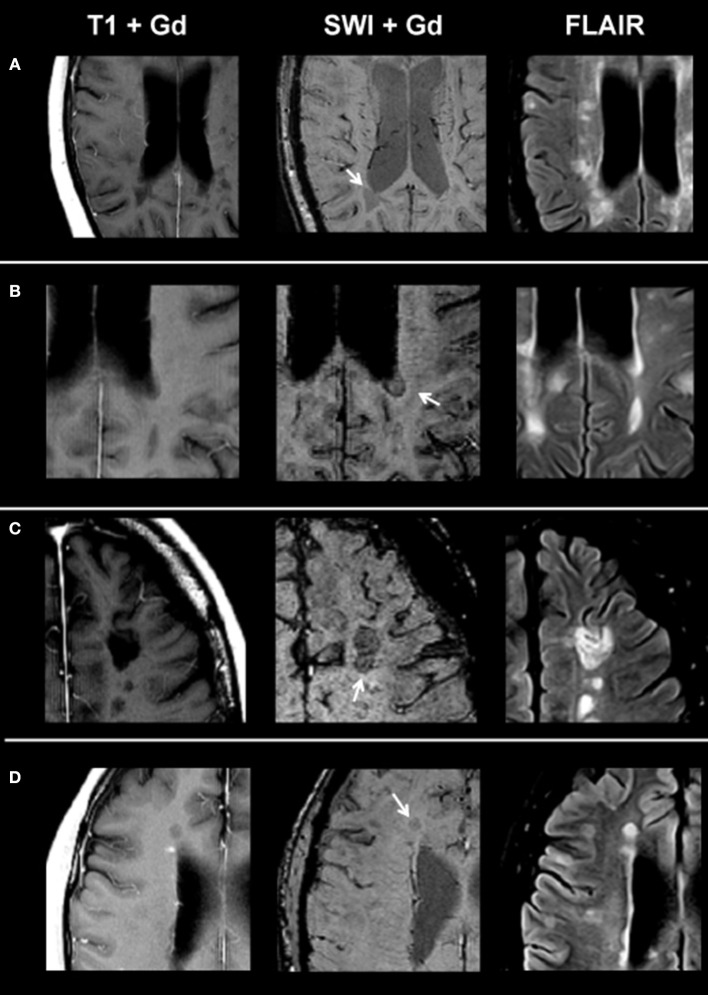
From left to right: representative post-contrast T1, post-contrast SWI, and FLAIR images of non-enhancing lesion subtypes. **(A)** Homogenously hypointense lesion, **(B)** ring-shaped distribution of susceptibility dots, **(C)** scattered distribution of susceptibility dots, and **(D)** lesion with a central dark region. SWI, susceptibility-weighted magnetic resonance imaging; FLAIR, fluid-attenuated inversion recovery.

On SWI, lesions in the non-enhancing lesion group 2 showed the following phenotypes: 374/1,246 lesions were homogenously hypointense without dark susceptibility dots, 162/1,246 showed ring-shaped distribution of susceptibility dots, 199/1,246 showed scattered distribution of susceptibility dots, 75/1,246 showed a central dot suggestive of a central vein, and 436/1,246 were not visible on SWI. Figure 2 demonstrates exemplary images of chronic lesion subtypes identified on SWI, and Table 2 provides an overview on signal characteristics.

**Table 2:** MRI characteristics of non-contrast-enhancing lesions.

	**SWI lesions**	**Lesions associated with veins on SWI**	**T1 hypointense lesions**
Homogenous hypointense lesions	374	172 (46%)	307 (82%)
Ring-shaped distribution of susceptibility dots	162	82 (51%)	138 (85%)
Scattered distribution of susceptibility dots	199	86 (43%)	145 (73%)
Lesions with a central dark region in SWI	75	26 (35%)	46 (61%)
Lesions not visible in SWI	436	–	279 (64%)
Total	1,246	366 (29%)	915 (73%)

*SWI, susceptibility-weighted magnetic resonance imaging*.

**Figure 3:**
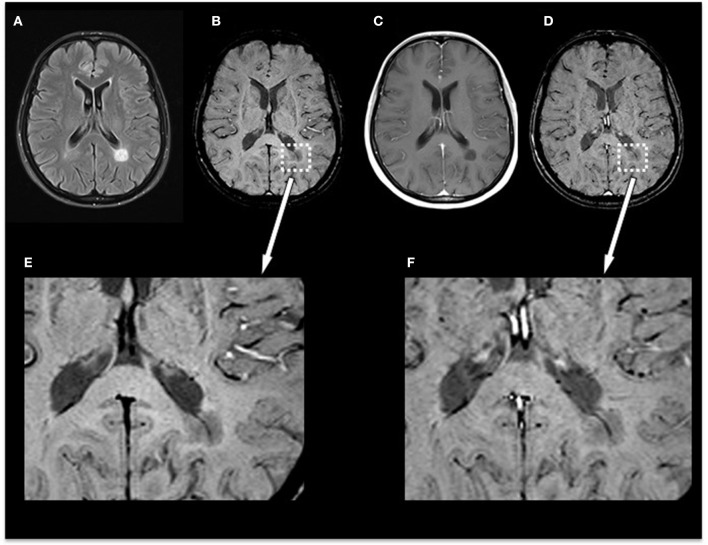
Exemplary MRI of a 44-year-old woman presenting with relapsing–remitting MS. **(A)** T2-FLAIR, **(B)** pre-contrast SWI and magnification **(E)**, **(C)** post-contrast T1-weighted (T1w), and **(D)** post-contrast SWI and magnification **(F)**. Conventional MRI demonstrates a chronic T1-hypointense lesion (“black hole”) that appears homogenously hypointense on SWI. Post-contrast SWI **(D)** and magnification **(F)** facilitated the visibility of the penetrating vein already seen in pre-contrast images but had no influence on the total number of veins detectable or diagnostic image quality and parenchymal signal alterations. MS, multiple sclerosis; FLAIR, fluid-attenuated inversion recovery; SWI, susceptibility-weighted magnetic resonance imaging.

In the subgroup of the 10 patients examined with SWI before and after contrast agent injection, we identified 35 non-enhancing lesions: 6/35 lesions were homogenously hypointense without dark susceptibility dots, 7/35 showed ring-shaped distribution of susceptibility dots, 5/35 showed scattered distribution of susceptibility dots, 2/35 showed a central dot suggestive of a central vein, and 15/35 were not visible on SWI. When comparing pre-contrast and post-contrast SWI, the contrast agent facilitated the visibility of small veins already seen in pre-contrast images but had no influence on the total number of veins detectable or diagnostic image quality and parenchymal signal alterations. Figure 3 demonstrates an example of an MS patient presenting a non-enhancing T1 hypointense lesion on pre-contrast and post-contrast SWI. In this subgroup, no CE lesions were present.

## Discussion

SWI is a relatively new MRI technique that has shown promise to provide additional information to established MRI methods in the diagnostic workup and monitoring of MS patients. We limited the analysis of SWI characteristics to lesions of at least 5 mm in diameter in order to be able to differentiate intrinsic lesion characteristics. As a consequence, the results obtained in this study may not or only partly apply to smaller lesions. The results of this study bring out several interesting points.

SWI can demonstrate features of lesions otherwise not visible. Central dots in keeping with central veins can be readily identified, which can help to confirm a typical MS feature as it has been described pathologically. This feature may have value for the differential diagnosis of MS and is currently being evaluated in regard to its potential to facilitate an early diagnosis (20). CE lesions represent a key feature in MS and are thought to demonstrate blood–brain-barrier permeability changes often facilitating the fulfillment of dissemination in time in the diagnosis of MS or demonstrating an ongoing disease activity despite immune treatment. To date, few studies have performed SWI after contrast application. A standard dose of gadolinium (0.1 mmol/kg) appears to provide optimal image quality for clinical application of contrast-enhanced SWI (15). An earlier study applied SWI before and after contrast agent injection in 31 healthy subjects. They found no signal contamination or quality degradation on SWI after application of a contrast agent (16). Other studies have demonstrated an increased detection of small veins in brain neoplasms (21) and MS (22–24). In our study, investigation of SWI after contrast injection facilitated the visibility of small veins already seen in pre-contrast SWI but had no influence on the total number of veins detectable in this subgroup of patients. We found in the CE lesion group that 50% of lesions are associated veins. This underlines one of the characteristics of MS lesions, and when considered together, a combination of (i) the location of a lesion, (ii) contrast enhancement, and (iii) a typical associated vein may have the potential to become highly informative and specific elements in the diagnosis of MS lesions. In this regard, CE SWI contains potentially valuable information for the characterization of focal MS pathology.

Furthermore, there has been recent interest in signs of smoldering active inflammation/presence of macrophages and demyelination, as this might be a pathological element that could contribute to slowly evolving clinical change (7, 25) that can be seen and may be of interest.

Using SWI, Haacke et al. detected different phenotypes of SWI phenomena in MS (10). This included lesions with scattered susceptibility hypointensities and lesions surrounded by a rim of hypointense signal suggestive of a ring-like shape of elevated iron levels. Our results are in line with those findings. From a histopathological point of view, lesions with a hypointense ring seem to indicate the presence of myelin-laden macrophages that may also participate in continuing smoldering inflammation (12). Current studies highlight the concept of “slowly expanding lesions” that may be best detectable on T1w MRI (12, 26). These lesions maintain some degree of inflammatory and myelin breakdown at the lesion edge after the demyelinating event (12). Various studies have demonstrated that iron accumulates within macrophages and microglia at the edges of these lesions, forming rims (8, 27). Using 7-T post-mortem MRI, a current study investigated iron accumulation at the edge of MS lesions. The authors demonstrated that a hypointense rim detected on SWI correlated histologically with iron accumulation in macrophages and microglia expressing the pro-inflammatory markers CD86 and p22phox at the edge of slowly expanding lesions, whereas non-rim lesions showed a tendency to shrink over time (12). The authors concluded that the presence of iron rims on SWI MRI might be a sign of progressive tissue injury and might serve as a marker of a disease activity in MS patients (12). We detected in 29% of lesions larger than 5-mm indications of smoldering inflammatory pathology in the form of scattered or peripheral susceptibility hypointensities. This number is larger than the absolute number of CE lesions in this study, which may indicate that the presence of this pathological change persists for a longer duration than the duration of contrast enhancement. This would be in line with the observation made in a follow-up study (12). To date, no comparative studies to other pathologies are available, and it is possible that hypointense rim lesions are specific for MS and not seen in other pathologies. This could be addressed in future studies.

One limitation of our study is the cross-sectional nature of the analysis and observations. We are aware that although no serial MRI data were available, this is certainly a limitation when trying to obtain information on dynamic phenomena of lesion evolution.

Our data add to the growing database of SWI in MS and point to a potential, useful additional information when integrating the information from CE and SWI MRI.

## Data Availability Statement

Anonymized data will be made available on request to researchers who meet the criteria for access to confidential data.

## Ethics Statement

The studies involving human participants were reviewed and approved by University Medicine Mannheim Ethics Committee. Patient consent was waived owing to the retrospective nature of the study and the lack of patient interaction.

## Author Contributions

PE and AG: conceptualization of the study, acquisition, analysis, interpretation of the data, drafting, and revising the manuscript. KP, KS, and MP: analysis and interpretation of the data and revising the manuscript.

### Conflict of Interest

PE has received travel allowance from Bayer Health Care. MP has a consultant relationship with Novartis, Merck, and Genentech/Roche; has received non-personal, institutional honoraria from Medac, Merck, Novartis, TEVA, and Genentech/Roche; and has research agreements with Bayer Health Care. AG has received honoraria for lecturing, travel allowance for attending meetings, and financial support for research from Bayer Schering, Biogen Idec, Merck Serono, Novartis, and TEVA Neurosciences and serves on the Editorial Board of Frontiers in Neurology—Applied Neuroimaging and Journal of Neuroimaging. The remaining authors declare that the research was conducted in the absence of any commercial or financial relationships that could be construed as a potential conflict of interest.
